# Impact of COVID-19 Pandemic on Elevated Anxiety Symptoms of Maintenance Hemodialysis Patients in China: A One-Year Follow-Up Study

**DOI:** 10.3389/fpsyt.2022.864727

**Published:** 2022-05-19

**Authors:** Honghong Lv, Junping Meng, Yang Chen, Feng Yang, Wen Wang, Guohua Wei, Jiaojiao Zhang, Huan Wang, Mengqiu Wang, Lu Zhou, Hongbao Liu

**Affiliations:** Department of Nephrology, Tangdu Hospital, The Air Force Military Medical University, Xi'an, China

**Keywords:** anxiety, COVID-19, risk factors, dialysis, kidney

## Abstract

Recent studies have shown that coronavirus disease 2019 (COVID-19) aggravates anxiety in patients with maintenance hemodialysis (MHD), but it is still unclear how long this adverse effect will last. This study aims to investigate the impact of COVID-19 on the elevated anxiety symptoms of MHD patients 1 year after the outbreak. Assessment of elevated anxiety symptoms was performed on patients with MHD during early COVID-19 (February 17-February 29, 2020) and 1-year follow-up (March 1-March 13, 2021), and a total of 100 patients had completed face-to-face questionnaires at the first and 1-year follow-up. At the beginning of the outbreak, 40% of the patients with MHD had anxiety symptoms [self-rating anxiety scale (SAS) score ≥ 50], and 11% (SAS score: 60–69) and 2% (SAS score ≥ 70) of the patients had moderate and severe anxiety symptoms, respectively. Multivariate analysis shows that possibility of unaccompanied transfer, possibility of family members or themselves being infected in a hospital, added body temperature monitoring during dialysis, and increased medical procedures are the risk factors in elevated anxiety symptoms during early COVID-19. At the 1-year follow-up, the incidence of anxiety symptoms in the same group of patients declined to 28%, and all the patients had mild anxiety symptoms (SAS score: 50–59), which is significantly lower than that of the early COVID-19 pandemic with statistically significant difference (*p* = 0.003). Increased protective measures taken by the medical staves were the only risk factor in elevated anxiety symptoms during the 1-year follow-up. This study shows that COVID-19 has a direct impact on the deterioration of anxiety symptoms in patients with MHD. With the changes of the requirements for COVID-19 prevention and control, as well as the enhancement of propaganda and education of the pandemic and psychological care, the severity and risk factors of anxiety symptoms in the patients with MHD are changing. Thus, targeted interventions are suggested to improve the psychological endurance of the patients with MHD.

## Introduction

Chronic kidney disease (CKD) is an important contributor to morbidity and mortality from non-communicable diseases. Many countries and medical institutions have focused on it to meet the UN's Sustainable Development Goal target of reducing premature mortality from non-communicable diseases by a third by 2030 (1). Maintenance hemodialysis (MHD), the most widely used treatment for end-stage renal disease (ESRD), has provided a life-sustaining treatment option for the vast majority of patients with ESRD (2). As of 2017, there were about 3 million patients with MHD worldwide, and the number is projected to reach 5.4 million by 2030 (1, 3). The increasing patients with MHD year by year incur significant financial burden to healthcare systems and ecological burden as well (4).

In addition to the physical symptoms, the patient's psychological burden is also the focus of the seven seas. Schouten et al. (5) showed that anxiety levels in patients with MHD were independently associated with an increased risk of death and 1-year hospitalization. The outbreak of a severe infectious disease called coronavirus disease 2019 (COVID-19) in December 2019 has caused global social and economic chaos (6, 7). The Chinese government took timely and orderly measures, such as quarantine, lockdown, maintaining physical distancing, and mask wearing, and incorporated the disease into the Class B infectious diseases stipulated by the Law of the People's Republic of China on Prevention and Control of Infectious Diseases and managed it according to the standard of Class A infectious diseases (8). Under the condition of closely controlling the travel of suspected cases, strengthening nucleic acid monitoring and encouraging people to inject COVID-19 vaccine, the situation in China has been basically brought under control, and economic and social activities have gradually been on track. During the early stage of the pandemic, special prevention and control measures increased the travel and medical access difficulties of patients with MHD. Various studies have shown that the incidence rate of anxiety and depressive symptoms in the patients with MHD increased during the pandemic, with the main influencing factors such as complications, comorbidity, social and cultural factors, etc. (9–22).

However, the overwhelming majority of current studies are limited to cross-sectional and lack of longitudinal-study. With the arrival of the normalized prevention and control management period of COVID-19 pandemic, understanding the changes in the psychological status of the patients with MHD during this period will be conducive to the adjustment strategy of psychological intervention for them. Therefore, this study was designed to focus on the short-term and long-term impacts of COVID-19 on elevated anxiety symptoms in the patients with MHD during the outbreak and in the 1-year follow-up, and explore the unique anxiety-related risk factors associated with the elevated anxiety symptoms.

## Methods

### Participants

This study was conducted in the Blood Purification Center of Tangdu Hospital, The Air Force Military Medical University. 20–30 patients with MHD are admitted to our center after ruling out ~10 patients with acute kidney injury per year. During the first questionnaire survey, 152 patients were receiving dialysis in our center. A total of 120 patients were enrolled under the inclusion and exclusion criteria from February 17 to February 29, 2020 instead of recruited consecutively. Inclusion criteria: (1) duration of hemodialysis, ≥ 3 months; (2) age, ≥18 years; no history of mental illness; (3) volunteered for a questionnaire survey. Exclusion criteria were developed as following: (1) patients with communication disorder; (2) long-term or recent use of psychotropic drugs; (3) those who refused or were unable to cooperate with the investigation for other reasons. According to observation in routine nursing work and communication with the patients and their families, no history of cognitive impairment was detected of all the patients. None of the patients had memory impairment, aphasia, mood changes, dementia, and other cognitive limitation-associated symptoms. This study was approved by the Internal Review and Ethics Boards of Tangdu Hospital, and the ethical approval assigned number of the study is TDLL-202201-06. This study was conducted in accordance with the Declaration of Helsinki. Due to the pandemic, the written informed consent was waived, and verbal informed consent was obtained from all the participants prior to enrollment in the assessment.

### Sample Size

The calculation of the sample size was based on the formula n = ${\text{Z}}_{\text{α}}^{2}\times$ pq/d^2^, where Z_α_ = 1.96 and α =0.05, and the estimated acceptable error range of the scale was 0.2 (23). According to previous studies conducted in USA (24), the proportion of MHD with elevated anxiety syndrome was estimated to be 53%. Considering that Xi'an is the transportation and trade hub with frequent turnover of staff in the northwest of China, we expanded the sample size by 20% so that atleast 107 questionnaires would be completed by the participants.

### Research Methods

The questionnaire survey was conducted by specially trained paramedics, and the questionnaire was issued to the patients on the spot. Patients were informed about the purpose and procedure of the survey and encouraged to describe their true feelings. After all the questions had been completed by the patients, the investigator signed on the questionnaire and sealed it up to ensure the patients' privacy and data reliability. The anxiety symptoms were assessed in two periods: early COVID-19 pandemic (February 17-February 29, 2020) and 1-year follow-up (March 1-March 13, 2021). The questionnaire consisted of the following three parts.

#### Demographic Data

The participants reported their gender, age, education, occupation, monthly income, duration of hemodialysis, medical insurance, etc., during the questionnaire period.

#### Anxiety Symptoms

The validated Chinese version of self-rating anxiety scale (SAS) (25) was used to assess levels of anxiety. The SAS scale was compiled by Zung (26), which is widely used in clinical and medical research as a norm-referenced scale to assess anxiety symptoms for the past few decades. The Cronbach α coefficient of the scale is 0.82. Zung's SAS scale has been translated into Chinese versions, with a high reliability coefficient for different populations in China (27, 28). There are 20 items in total, with 1–4 points for each item and 15 forward scores and 5 reverse scores, respectively. The rough score is the sum of the scores of the 20 items, and the standard score (Chinese norm) = rough score × 1.25 (the result is an integer). According to the Chinese norm results, the scores for no anxiety, mild anxiety, moderate anxiety, and severe anxiety were < 50, 50–59, 60–69, and ≥70, respectively; thus, it can be seen that the SAS score is positively correlated with the degree of anxiety, that is, the higher score, the severer anxiety.

#### Self-Designed Special Factors That May Influence Anxiety Syndrome During the Pandemic

The respondents reported 10 self-reported questions related to the COVID-19 pandemic: possibility of unaccompanied transfer, being in a designated hospital for COVID-19 treatment, travel difficulties, increased protective measures taken by the medical staves, increased medical procedures, decreased communication between patients-patients/patients-family members, possibility of the family members or themselves being infected in the hospital, added body temperature monitoring during dialysis, possibility of emergency dialysis, and desire to reduce the frequency of dialysis. The scale is scored based on a five-point Likert scale (Never = 1 to always = 5).

### Statistics

The data were analyzed statistically by SPSS 22.0 (BM Corp). The continuous variables were expressed as the mean ± standard deviation (SD), and Student's *T*-tests or Kruskal–Wallis tests were used for inter-group comparison. The classification variables were expressed as frequencies and percentages, and chi-square tests were used for inter-group comparison. Univariate linear regression models were adopted to analyze the correlation between SAS scores and possible predictor variables. The variables included demographic data and self-reported questions associated with the COVID-19 pandemic. The variables with statistical significance were selected based on univariate analyses (set as *p* < 0.1), and multivariate linear regression analyses were used to explore the potential risk factors for predicting SAS scores during the early COVID-19 pandemic and the 1-year follow-up. *p* < 0.05 was considered statistically significant.

## Results

### Survey Respondents

**Figure 1:**
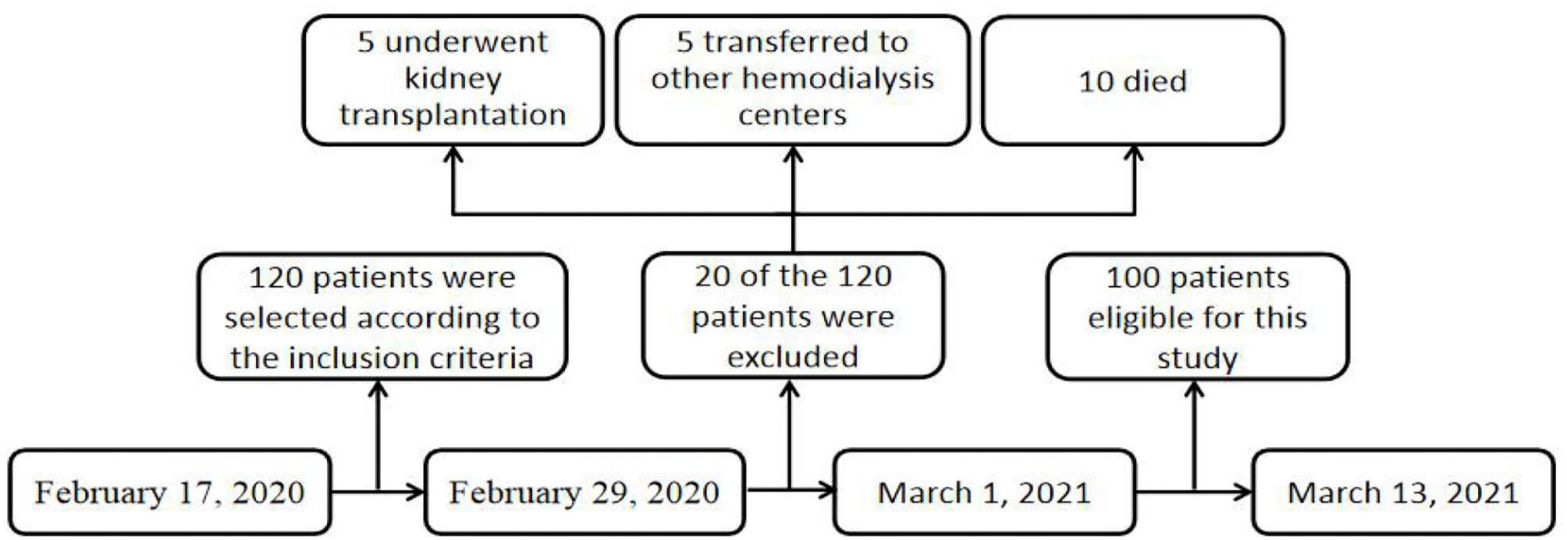
A flow diagram of study design.

The research process is shown in Figure 1. A total of 120 patients with MHD completed the first questionnaire survey. In the 1-year follow-up, 20 of the 120 patients were excluded: five underwent kidney transplantation, five transferred to other hemodialysis centers, and 10 died. The remaining 100 respondents completed the follow-up questionnaire. Of the 100 patients, 45% were female, aged 25–80 years. Most patients (78%) received senior or less education. About 25% of the patients have full-time or part-time jobs. The duration of dialysis in 59% of the patients was more than 3 years. About 95% of the patients were covered by national medical insurance (employee or resident). The patients with MHD with causes of primary glomerulonephritis (43%), diabetes (28%), and hypertension (20%) account for 91% of all the patients.

### Impact of Different Pandemic Periods on Elevated Anxiety Symptoms in Patients With MHD

Anxiety symptom profiles among patients with MHD during the early COVID-19 and at 1-year follow-up are shown in Table 1. The SAS scores of 100 patients with MHD who completed both questionnaires were (47.81 ± 9.57) and (45.23 ± 6.30) in the early stage of the outbreak and 1-year follow-up, respectively, with statistically significant differences (*p* = 0.026). In the early stage of the pandemic, 40% of the patients had a SAS score of ≥ 50, indicating the presence of elevated anxiety symptoms. Mild, moderate, and severe anxiety syndromes were 27 cases (67.5%), 11 cases (27.5%), and two cases (5%), respectively. During the 1-year follow-up 28% of the same group of the patients had elevated anxiety symptoms (all mild), which was significantly lower than that in the early stage of the pandemic (*p* = 0.003).

**Table 1:** Anxiety status of hemodialysis patients in different periods of the pandemic.

**Variables**	**Early COVID-19 pandemic (*n* = 100)**	**One-year follow-up (*n* = 100)**	***t-*value or χ^2^**	***p-*value**
SAS score (mean ± SD)	47.81 ± 9.57	45.23 ± 6.30	2.252	0.026
<50, *n* (%)	60 (60 %)	72 (72 %)	14.109	0.003
50~59, *n* (%)	27 (27 %)	28 (28 %)		
60~69, *n* (%)	11 (11 %)	0 (0 %)		
≥70, *n* (%)	2 (2 %)	0 (0 %)		

### Demographic Characteristics of Patients With MHD With and Without Elevated Anxiety Symptoms During Early COVID-19 and at 1-Year Follow-Up

Patients with MHD with and without elevated anxiety symptoms (SAS score of ≥ 50) were compared on multiple variables during early COVID-19 and at 1-year follow-up (Table 2). The education level was found to be different between the two groups (χ^2^ = 6.433, *p* = 0.040) during the early COVID-19 pandemic. However, there was no statistical difference in the 1-year follow-up. Surprisingly, among the patients with MHD with bachelor degree or above, two patients who had no anxiety symptoms initially developed mild anxiety symptoms at 1-year follow-up.

**Table 2:** Demographic characteristics of hemodialysis patients with and without elevated anxiety symptoms during the early COVID-19 pandemic and at 1-year follow-up (*n* = 100).

	**Early COVID-19 pandemic**			**One-year follow-up**		
**Characteristics**	**SAS score <50 (*n* = 60)**	**SAS score ≥50 (*n* = 40)**	***p-*value**	**SAS score <50 (*n* = 72)**	**SAS score ≥50 (*n* = 28)**	***p*-value**
**Female**, *n* (%)	26 (43.3%)	19 (47.5%)	0.682	34 (47.2%)	11 (39.3%)	0.474
**Age** (years), *n* (%)			0.347			0.289
<45	16 (26.7%)	6 (15%)		13 (18.1%)	8 (28.6%)	
45–59	23 (38.3%)	16 (40%)		27 (37.5%)	12 (42.9%)	
≥60	21 (35%)	18 (45%)		32 (44.4%)	8 (28.6%)	
**Education**, *n* (%)			0.040			0.428
Primary or less	14 (23.3%)	18 (45%)		21 (29.2%)	11 (39.3%)	
Junior or senior	29 (48.3%)	17 (42.5%)		36 (50.0%)	10 (35.7%)	
University degree or above	17 (28.3%)	5 (12.5%)		15 (20.8%)	7 (25.0%)	
**Occupation**, *n* (%)			0.638			0.505
Employment	17 (28.3%)	8 (20%)		17 (23.6%)	8 (28.6%)	
Retired	26 (43.3%)	19 (47.5%)		35 (48.6%)	10 (35.7%)	
Unemployment or others	17 (28.3%)	13 (32.5%)		20 (27.8%)	10 (35.7%)	
**Monthly income (CNY)**, *n* (%)			0.681			0.116
≤3,000	16 (26.7%)	15 (37.5%)		25 (34.7%)	6 (21.4%)	
3,001–5,000	19 (31.7%)	12 (30%)		22 (30.6%)	9 (32.1%)	
5,001–10,000	20 (33.3%)	10 (25%)		22 (30.6%)	8 (28.6%)	
>10,000	5 (8.3%)	3 (7.5%)		3 (4.2%)	5 (17.9%)	
**Duration of hemodialysis (months)**, *n* (%)			0.550			0.276
3 −36	25 (41.7%)	16 (40.0%)		19 (26.4%)	4 (14.3%)	
37–72	20 (33.3%)	17 (42.5%)		27 (37.5%)	15 (53.6%)	
73–108	8 (13.3%)	2 (5.0%)		14 (19.4%)	7 (25.0%)	
109 months or more	7 (11.7%)	5 (12.5%)		12 (16.7%)	2 (7.1%)	
**Medical insurance**, *n* (%)			0.406			0.319
6% Self-pay (Employee)	40 (66.7%)	22 (55%)		46 (63.9%)	16 (57.1%)	
40% Self-pay (Resident)	18 (30%)	15 (37.5%)		24 (33.3%)	9 (32.1%)	
100% Self-pay	2 (3.3%)	3 (7.5%)		2 (2.8%)	3 (10.7%)	
**ESRD etiology**, *n* (%)			0.418			0.222
Primary glomerulonephritis	28 (46.7%)	15 (37.5%)		35 (48.6%)	8 (28.6%)	
Diabetes	15 (25.0%)	13 (32.5%)		18 (25.0%)	10 (35.7%)	
Hypertension	10 (16.7%)	10 (25.0%)		12 (16.7%)	8 (28.6%)	
Others	7 (11.7%)	2 (5.0%)		7 (9.7%)	2 (7.1%)	

*SAS, self-rating anxiety scale; CNY, Chinese Yuan; ESRD, end-stage renal disease*.

### Predictive Factors of Anxiety Symptoms During Early COVID-19 and at the 1-Year Follow-Up by Multivariate Linear Regression Analyses

Univariate linear regression analyses were performed, and the SAS score of early COVID-19 was set as a dependent variable, with predictors including gender, age, education, occupation, monthly income, duration of hemodialysis, medical insurance, and 10 self-reported questions associated with the COVID-19. The results show that elevated anxiety symptoms are relevant to education and eight self-reported questions (Table 3): possibility of unaccompanied transfer, being in a designated hospital for COVID-19 treatment, travel difficulties, increased protective measures taken by the medical staves, increased medical procedures, possibility of family members or themselves being infected in the hospital, added body temperature monitoring during dialysis, and possibility of emergency dialysis. These factors were selected as candidate predictors for multivariate linear regression analyses. The possibility of unaccompanied transfer, possibility of family members or themselves being infected in the hospital, added body temperature monitoring during dialysis, and increased medical procedures are risk factors for elevated anxiety symptoms during the early COVID-19 pandemic (Table 4). The R^2^ of the multivariate linear regression was 0.268. In the 1-year follow-up, linear regression analyses show that increased protective measures taken by the medical staves was the only predictor for the SAS score (Table 5).

**Table 3:** Univariate linear regression analysis of factors predictive of elevated anxiety symptoms during the early COVID-19 pandemic.

**Characteristics**	**B**	**S.E**.	***t*-value**	***p*-value**	**95% C.I**.
Gender (Male/Female)	2.244	1.920	1.169	0.245	−1.566; 6.055
Age (years)	1.312	1.255	1.045	0.298	−1.179; 3.802
Education	−2.904	1.288	−2.254	0.026	−5.461; −0.347
Occupation	1.223	1.294	0.945	0.347	−1.346; 3.791
Monthly income (CNY)	−0.729	1.007	−0.724	0.471	−2.728; 1.270
Duration of hemodialysis (months)	−0.511	0.968	−0.528	0.599	−2.432; 1.410
Medical insurance	1.321	1.581	0.835	0.405	−1.817; 4.459
Possibility of unaccompanied transfer	2.526	0.624	4.050	0.000	1.288;3.764
Being in a designated hospital for COVID-19 treatment	1.984	0.627	3.165	0.002	0.740; 3.227
Travel difficulties	1.985	0.791	2.510	0.014	0.416; 3.555
Increased protective measures taken by the medical staves	1.663	0.689	2.415	0.018	0.297;3.030
Increased medical procedures	1.943	0.730	2.662	0.009	0.495;3.392
Decreased communication between patients-patients /patients- family members of patients	1.468	1.495	0.982	0.328	−1.499;4.436
Possibility of family members or themselves being infected in the hospital	1.853	0.624	2.970	0.004	0.615; 3.090
Added body temperature monitor during dialysis	1.865	0.705	2.645	0.010	0.466; 3.264
Possibility of emergency dialysis	1.358	0.659	2.061	0.042	0.050; 2.666
Desire to reduce the frequency of dialysis	0.588	0.750	0.784	0.435	−0.900; 2.075

*B, coefficient; S.E., standard error of estimated coefficient; C.I., confidence interval*.

**Table 4:** Multiple linear regression analysis of factors independently predictive of elevated anxiety symptoms during the early COVID-19 pandemic.

**Variables**	**B**	**S.E**.	***t*-value**	***p*-value**	**95% C.I**.
Possibility of unaccompanied transfer	1.778	0.622	2.858	0.005	0.543; 3.014
Possibility of family members or themselves being infected in the hospital	1.356	0.582	2.330	0.022	0.200; 2.511
Added body temperature monitor during dialysis	1.509	0.646	2.335	0.022	0.226; 2.792
Increased medical procedures	1.508	0.686	2.197	0.030	0.145; 2.871

*B, coefficient; S.E., standard error of estimated coefficient; C.I., confidence interval. R^2^ = 0.268*.

**Table 5:** Univariate linear regression analysis of factors predictive of elevated anxiety symptoms at 1-year follow-up.

**Characteristics**	**B**	**S.E**.	***t*-value**	***p*-value**	**95% C.I**.
Gender (Male/Female)	0.107	1.272	0.084	0.933	−2.418; 2.632
Age (years)	−0.285	0.835	−0.342	0.733	−1.943; 1.372
Education	0.477	0.868	0.550	0.584	−1.246; 2.200
Occupation	−0.332	0.855	−0.388	0.699	−2.028; 1.365
Monthly income (CNY)	1.295	0.652	1.988	0.050	0.002; 2.588
Duration of hemodialysis (months)	0.397	0.654	0.607	0.545	−0.902; 1.696
Medical insurance	1.046	1.072	0.976	0.332	−1.082; 3.175
Possibility of unaccompanied transfer	1.029	0.647	1.591	0.115	−0.254; 2.313
Being in a designated hospital for COVID-19 treatment	0.519	0.689	0.753	0.453	−0.849; 1.886
Travel difficulties	0.585	0.650	0.901	0.370	−0.704; 1.875
Increased protective measures taken by the medical staves	1.663	0.758	2.194	0.031	0.159; 3.167
Increased medical procedures	0.333	0.624	0.534	0.595	−0.905; 1.572
Decreased communication between patients-patients /patients- family members	0.667	0.677	0.985	0.327	−0.676; 2.011
Possibility of family members or themselves being infected in the hospital	0.242	0.576	0.421	0.675	−0.900; 1.384
Added body temperature monitoring during dialysis	0.407	0.636	0.640	0.524	−0.855; 1.668
Possibility of emergency dialysis	0.600	0.562	1.068	0.288	−0.514; 1.714
Desire to reduce the frequency of dialysis	−0.259	0.582	−0.445	0.658	−1.413; 0.896

*B, coefficient; S.E., standard error of estimated coefficient; C.I., confidence interval*.

## Discussions

The COVID-19 outbreaks in late 2019 has raged ever since, which had forced patients with MHD to face numerous pandemic-related factors and experienced unprecedented challenges (11–13, 17). This study investigates the sustained effects of COVID-19 on elevated anxiety syndrome in patients with MHD and its main influencing factors. Our findings show that the incidence of elevated anxiety symptoms in the patients with MHD decreased from 40% during early COVID-19 to 28% at the 1-year follow-up. Multivariate analysis shows that the risk factors for the deterioration of anxiety symptoms changed from the possibility of unaccompanied transfer, the possibility of family members or themselves being infected in the hospital, added body temperature monitoring during dialysis, and increased medical procedures to increased protective measures taken by the medical staves; that means, with the change of the requirements for COVID-19 prevention and control, the severity and risk factors of anxiety symptoms in the patients with MHD are also changing.

The majority of patients with MHD are elderly and always have complications such as diabetes, hypertension, and cardiovascular diseases, with lower immune function than general population, and are prone to dialysis-related anxiety disorders. Studies have shown that elevated anxiety symptoms are closely related to low life quality and high mortality in the patients with MHD, and have adverse effects on long-term treatment (5, 29). During the early COVID-19 pandemic, many countries implemented nationwide lockdowns in line with containment policies. However, the patients with MHD need to commute between home and a hospital regularly by public transportation, and stay with doctors, nurses or other patients in a shared space for at least 8–12 h per week due to the specificity of the dialysis treatment, which makes the patients cannot help but to face a complex medical and social environment, resulting in an increased concern about the transmission of COVID-19 by medical staff, infection of themselves or their family members and the surrounding environment, and serious reduction in socialization (10). As susceptible populations of anxiety, under the combined action of above factors, fear and apprehension induced by COVID-19 may aggravate the psychological disorders of the patients with MHD (10, 30–33), resulting in the aggravation of anxiety symptoms, manifested as shortness of breath, accelerated heart rate, pain, nervousness, and insomnia (10, 30).

Anxiety symptoms and disorders, as key research topics, were emphasized in the Kidney Disease: Improving Global Outcomes (KDIGO) Controversies Conference in Supportive Care (34). Although several studies have evaluated the association between anxiety and ESRD, the incidence of anxiety varies greatly due to differences in patient populations, study design, geographic environment, and diagnostic tools. Various scales were conducted to assess the anxiety syndrome in dialysis patients (5, 9, 35–42). Three studies using SAS as diagnostic tools have shown that 34.89%-44.90% of the patients with MHD have elevated anxiety symptoms (9, 38, 39). Several studies investigated that monthly income, education, work situation, medical insurance, dialysis duration, epidemic propaganda, and physical condition are closely associated with the elevated anxiety syndrome (9, 10).

Our research shows that 40% of the patients with MHD exist elevated anxiety symptoms (a SAS score of ≥ 50) in the early COVID-19 pandemic, and the severity of nearly 1/3 is moderate or higher (a SAS score of ≥ 60). The elevated anxiety syndrome of the patients with MHD during the outbreak is inseparable from the severity of the epidemic, the inconvenience and pressure caused by prevention and control management (10, 11, 14, 22). The strict social isolation measures made it difficult for the patients and their families to travel, which caused increased worry about unaccompanied transfer in patients with MHD. Large quantities of the patients with MHD and their families flowed between hospital and families, which increased the risk of spreading the pandemic and caused concern among the patients. Frequent temperature monitoring during the pandemic may also cause some patients to worry about a sudden rise of abnormal body temperature and subsequent isolation, resulting in tension and elevated anxiety symptoms. The increased medical procedures due to the strict implementation of the COVID-19 prevention and control regulations have increased the waiting time for treatment and the total outside time, which may cause the risk of aggravation and infection increased, therefore excerbating the anxiety of some patients. Studies have shown that workload is also one of the factors associated with anxiety symptoms among frontline medical staff during the pandemic (43). However, in the patients with MHD, consistent with other pieces of research, this study shows no significant difference in elevated anxiety symptoms between employed, retired, and non-employed patients with MHD (11, 40). Consequently, focusing on the specific factors related to COVID-19 and taking appropriate interventions were key to managing MHD patients during the follow-up period.

During the first questionnaire survey, China enacted and implemented strict prevention and control measures across the country, such as wearing masks, quarantining at home, and restricting hospital companionship, etc. Local government also applied a series of management protocols for blood purification centers, such as setting up prevention and treatment teams, formulating new infectious disease management regulations, dividing functional areas in blood purification centers. A series of targeted interventions were taken by the medical staves of our center to strengthen the psychological care and propaganda and education of the patients with MHD with the help of Internet tools in the following 1 year. One year later, a follow-up survey was carried out on the same group of patients. The results show that the incidence rate of elevated anxiety symptoms in the patients with MHD dropped from 40 to 28%, and all of them were mild anxiety symptoms (SAS scores: 50–59). At the same time, the factors inducing elevated anxiety symptoms have also changed from insufficient understanding of the disease, changing of lifestyle, and insufficient communication during the early COVID-19 pandemic to specific factors - increased protective measures taken by the medical staves. The statistics above show that the alleviation of anxiety syndrome is closely related to elimination or weakening of the COVID-19-related factors at the initial stage of the pandemic. On the basis of the stable control of the pandemic nationwide, refined interventions effectively guaranteed the safety of dialysis, stabilized the psychological status of patients, and alleviated anxiety syndrome. Firstly, effective prevention and control measures prevented the spread of the pandemic among the patients with MHD and provided a safe dialysis environment, which ensured the smooth progress of dialysis, and provided a powerful and forceful guarantee for the life safety of the patients. Secondly, the propaganda and education ensured that the patients mastered the correct method of preventing infection and enabled the patients to correctly recognize and rationally treat the pandemic, the psychological care dredged the patients' negative emotions, and enhanced their belief in overcoming the epidemic.

## Limitations of the Study

To the best of our knowledge, there is still no follow-up study of anxiety among the patients with MHD during the COVID-19 pandemic. However, there are still several limitations in this study. Primarily, this study was a single-center study, which still needs support and validation in large-scale clinical practice. In the next place, risk factors related to anxiety can be considered from the following four aspects in general: patient-related factors (demographics, socioeconomic status, dietary habits, and physical activity), medical condition-related factors (the primary cause of ESRD, laboratory abnormalities, interdialytic weight and related symptoms, psychiatric condition, comorbidity index, complications, etc.), therapy-related factors (medication, dialysis, and diet), and psychosocial-related factors (40). On account of observing the condition of elevated anxiety symptoms in the same group of the patients with MHD during different periods of the COVID-19 pandemic, this study only screened the basic information of the patients and COVID-19-related factors, while medical condition-related factors and therapy-related factors were not evaluated. Thirdly, it is regrettable that there is still no consensus on the gold standard tool for assessing anxiety in the patients with MHD for the reason of each instrument has inherent strengths and limitations; thus, only SAS was selected instead of multiple questionnaires to assess the anxiety symptoms of the patients with MHD just to avoid the complexity of MHD-related care in patient consultations. Fourthly, since the sudden outbreak of COVID-19 and even still raging all over the world, it is hard to compare the situation of the patients with MHD on the anxiety symptoms in the non-pandemic so that only the initial and 1-year follow-up of the patients with MHD on anxiety symptoms during the epidemic is compared in this study. Further study is still expected when the epidemic situation is completely eliminated to consummate the comparison.

## Conclusions

In conclusion, the results may be considered unique since the questionnaire survey was a follow-up study and conducted during an unprecedented period of COVID-19 among the patients with MHD. The anxiety-related factors of the patients with MHD changed with the condition of the pandemic and the effective intervention, and the level of anxiety symptoms also changed accordingly. Thus, we should pay more attention to the psychological status of the patients with MHD, and take timely effective measures to ensure smooth long-term treatment, improve service quality, and promote the management level.

## Data Availability Statement

The raw data supporting the conclusions of this article will be made available by the authors, without undue reservation.

## Author Contributions

HLv was responsible for the conception and design of the study and writing of the manuscript. FY, WW, GW, MW, JZ, and HW revised the manuscript critically for important intellectual content. JM and YC contributed to acquisition, analysis, and interpretation of data. LZ and HLiu finalized the content to be published and be accountable for all the aspects of the work in ensuring that the questions related to the accuracy or integrity of any part of the work are appropriately investigated and resolved. All the authors reviewed the manuscript critically and approved the final version.

## Funding

This work was supported by grants from Subject Platform and Technology Innovation Development Foundation of Tangdu Hospital (2019QYTS003, 2020XKPT014, and 2021QYJC-001) and Nursing Research Foundation of Tangdu Hospital (TDHLKY-2019-05).

## Conflict of Interest

The authors declare that the research was conducted in the absence of any commercial or financial relationships that could be construed as a potential conflict of interest.

## Publisher's Note

All claims expressed in this article are solely those of the authors and do not necessarily represent those of their affiliated organizations, or those of the publisher, the editors and the reviewers. Any product that may be evaluated in this article, or claim that may be made by its manufacturer, is not guaranteed or endorsed by the publisher.

## Abbreviations

COVID-19: Coronavirus disease 2019
MHD: Maintenance hemodialysis
MPD: Maintenance peritoneal dialysis
SAS: Self-rating anxiety scale
KDIGO: Kidney disease: improving global outcomes
ESRD: End-stage renal disease.
